# Oxytocin, vasopressin, and Williams syndrome: epigenetic effects on abnormal social behavior

**DOI:** 10.3389/fgene.2015.00028

**Published:** 2015-02-17

**Authors:** Brian W. Haas, Alicia K. Smith

**Affiliations:** ^1^Department of Psychology, University of Georgia, Athens, GA, USA; ^2^Interdisciplinary Neuroscience Graduate Program, University of Georgia, Athens, GA, USA; ^3^Department of Psychiatry and Behavioral Sciences, School of Medicine, Emory University, Atlanta, GA, USA

**Keywords:** Williams syndrome, genetics, *OXTR*, oxytocin, social behavior

## Abstract

Williams syndrome (WS) is a condition caused by a deletion of ∼26–28 genes on chromosome 7q11.23 often characterized by abnormal social behavior and disrupted oxytocin (OT) and vasopressin (AVP) functioning. The observation that individuals with WS exhibit OT and AVP dysregulation is compelling. There is currently a lack of evidence that any of the genes typically deleted in WS have any direct effect on either OT or AVP. In this perspective article, we present a novel epigenetic model describing how DNA methylation may impact the expression of key genes within the OT and AVP systems, which may ultimately influence the social behavior observed in WS. We draw support from data pooled from a prior empirical research study (Henrichsen et al., 2011), demonstrating that *OXTR* is overexpressed in WS. These preliminary findings may create new opportunities to target the OT and AVP systems with the specific goal of improving outcomes in WS and other psychiatric conditions.

## OXYTOCIN, VASOPRESSIN, AND SOCIAL BEHAVIOR

Oxytocin (OT) and vasopressin (AVP) are neurohypophysial hormones primarily synthesized within the hypothalamus of the brain. Both OT and AVP are involved in the pathophysiology for many psychiatric conditions that include autism, depression, and anxiety (Meyer-Lindenberg et al., 2011; Ebstein et al., 2012). For example, peripheral measures of OT are reduced in autism (Modahl et al., 1998), and OT administration may reduce the severity of symptoms in autism (Guastella et al., 2010). Accordingly, there are clinical trials currently underway designed to test the efficacy of OT administration on improving sociability and communication in children and teens with autism. In spite of a mass of empirical data showing that the OT and AVP systems are perturbed in neurodevelopmental and mood disorders, the mechanisms that lead to disrupted OT and AVP function are currently unclear.

## REGULATION OF OXYTOCIN AND VASOPRESSIN EXPRESSION

One way to improve the understanding of the OT and AVP systems is to investigate genes that influence the function of OT and AVP. Some genes that impact OT and AVP function include *OXTR* and *AVPR1A*, which encode the OT and AVP receptors, respectively, throughout the brain and body (Kimura et al., 1992; Inoue et al., 1994; Koshimizu et al., 2012). Expression profiles and/or commonly occurring variants [e.g., single nucleotide polymorphisms (SNPs)] of *OXTR* and *AVPR1A* are associated with individual differences in human social behavior, emotion processing, and many psychiatric conditions. Thanseem et al. (2012) showed reduced expression of *OXTR* in autism. SNPs of *OXTR* are associated with brain reactivity to emotional stimuli (Haas et al., 2013) and behavioral measures of empathy (Rodrigues et al., 2009), trust (Krueger et al., 2012), and emotion recognition (Lucht et al., 2013). Variation of *AVPR1A* expression is associated with social behavior in prairie voles (Barrett et al., 2013). Commonly occurring polymorphic microsatellite repeats of *AVPR1A* are associated with brain reactivity to emotional stimuli (Zink and Meyer-Lindenberg, 2012) and behavioral measures of generosity (Avinun et al., 2011), pair bonding (Walum et al., 2008), and harm avoidance (Meyer-Lindenberg et al., 2008). Together, this research indicates that *OXTR* and *AVPR1A* influence the function of the OT and AVP systems, and thus affect social behavior and emotion processing. It is currently unknown however, what mechanisms contribute to altered expression of *OXTR* and *AVPR1A* genes. A missing component within the puzzle that explains altered OT and AVP functioning is a factor that impacts the expression of genes within the OT and AVP systems.

In addition to DNA sequence variation, epigenetic mechanisms can also influence OT and AVP expression. The field of epigenetics seeks to understand how structural modifications to DNA or its protein scaffolding, such as histone modification or DNA methylation, regulate the expression of specific genes. Many contemporary models of psychiatric illness embrace the concept that epigenetic modifications influence the onset and severity of specific symptoms (Smith et al., 2014). Thus, by understanding the mechanisms that influence the activity and expression of genes, a more complete, mechanistic view of many forms of psychiatric illness can be reached. In this perspective article, we present a theoretical epigenetic model describing how a specific set of social behaviors may be influenced by epigenetic processes within the OT and AVP systems.

## WILLIAMS SYNDROME, A UNIQUE WINDOW TO GENETIC INFLUENCES ON THE SOCIAL AND EMOTIONAL BRAIN

Williams syndrome (WS) is a model system for understanding genetic influences on social-emotional processing (Jarvinen-Pasley et al., 2008). WS is caused by a deletion of ∼26–28 genes on chromosome 7q11.23 and is often paired with a distinctive social-emotional profile characterized by reduced social inhibition, an increased affinity toward attending to faces and a reduced sensitivity to fear-related social stimuli (Martens et al., 2008; Haas and Reiss, 2012). Because WS is caused by a genetic deletion, studying this condition provides a rare opportunity to explore gene-brain-social behavior associations in humans (Hoeft et al., 2014). More specifically, by studying WS, evidence that specific genes impact human social behavior and emotion processing can be obtained.

Several aspects of the WS social-emotional phenotype mirror those typically associated with other neurodevelopmental condition, such as autism. One characteristic behavior associated with WS is the tendency to be less socially inhibited and more trusting of others as compared to typically developing (TD) controls. For example, individuals with WS are more willing to approach strangers (Dodd et al., 2010) and rate facial expressions as more trustworthy or approachable (Jones et al., 2000; Martens et al., 2012) relative to controls. People with WS also tend to exhibit an abnormally high amount of attention toward social stimuli such as faces. Studies using eye-tracking show that people with WS fixate on faces longer (Riby and Hancock, 2008, 2009) and are slower to disengage their gaze once fixated on eyes (Porter et al., 2010) or a face (Riby et al., 2011) as compared to controls. Lastly, individuals with WS tend to be less sensitive to fear-related social stimuli than controls (Plesa-Skwerer et al., 2009). Together, these findings suggest that biological systems involved in social-emotional processing are affected by the deletion of genes in WS.

Several neuroimaging studies have demonstrated that key brain regions involved in social-emotional processing are functionally and structurally abnormal in WS. For example, functional neuroimaging research shows that individuals with WS exhibit larger areas of brain activation (within the fusiform face area) when processing faces compared to controls (Golarai et al., 2010). In addition, individuals with WS exhibit reduced amygdala response to fearful facial expressions (Meyer-Lindenberg et al., 2005), and increased amygdala response to happy facial expressions (Haas et al., 2009). Structurally, individuals with WS exhibit increased amygdala volume (Martens et al., 2009; Haas et al., 2014b), and increased fractional anisotropy within the amygdala and fusiform gyrus (Haas et al., 2014a) relative to TD controls. These findings suggest that the genes deleted in WS may influence the development of the human social and emotional brain.

Recently, compelling evidence has emerged that the OT and AVP systems are perturbed in WS (Dai et al., 2012). Dai et al. (2012) collected blood plasma levels from a sample with WS and TD controls at baseline and at multiple time points following a positive emotional intervention (music), and a negative physical stressor (cold). The results showed that OT and AVP levels were increased in WS at baseline, and in response to psychological manipulations (music and cold). Additionally, WS was associated with greater variability in OT and AVP response as compared to controls. These findings support the hypothesis that the regulation of OT and AVP is altered in WS.

It is currently unknown what mechanisms impact altered OT and AVP functioning in WS. There is currently no evidence that genes that code for the synthesis, transmission or signal transduction of OT or AVP are located within the “WS classic deletion region.” This presents an intriguing question. Are there other (possibly indirect) mechanisms through which the deleted genes in WS can impact the function of the OT and AVP systems?

## *OXTR* AND *AVPR1A* EXPRESSION

**FIGURE 1:**
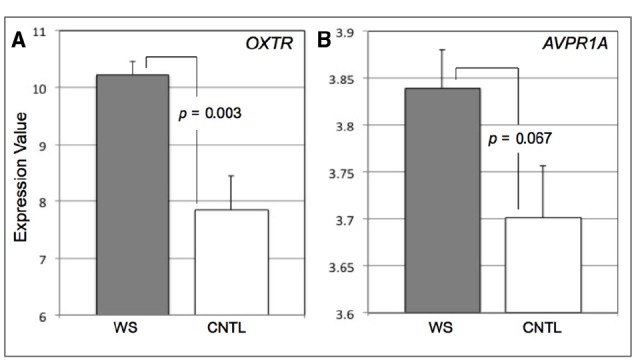
**Bar graphs displaying expression values for *OXTR* (A) and *AVPR1A* (B) in Williams syndrome (WS) and healthy controls (CNTL).** Error bars represent standard error from the mean.

One factor that may impact OT and AVP functioning in WS is altered expression of *OXTR* and *AVPR1A*. Prior studies have shown abnormities in gene expression in WS, but have not focused specifically on genes within the OT and AVP systems. We evaluated gene expression data from a previously published study on gene expression in WS (Henrichsen et al., 2011), using the Gene Expression Omnibus (GEO)^1^, a public genomics data repository. Data were selected and downloaded using GEO2R^2^, an interactive web platform used to compare samples across experimental conditions (GSE16715). Based on prior evidence that OT and AVP are dysregulated in WS (Dai et al., 2012), we compared expression of *OXTR* (ID: 206825_at) and *AVPR1A* (ID: 206250_x_at) between eight WS patients (mean age = 4.87 years; standard deviation = 2.10) and nine age and sex matched healthy controls (CNTL; *M* = 4.77; SD = 1.79; Figure 1). Within the GEO2R, data for these two transcripts were extracted and analyzed using default settings (Benjamini–Hochberg—false discovery rate and auto-detect for log transformation).

We found that the WS subjects exhibited greater expression of *OXTR* (*M* = 10.22, SD = 0.68) compared to CNTLs (*M* = 7.86, SD = 1.79) (*t* = 3.51, *p* = 0.003, 95% Confidence Interval = 0.93–3.81) (Figure 1A). Though the mean expression of *AVPR1A* was higher in WS, there was no statistical difference between the WS (*M* = 3.84, SD = 0.11) and CNTL (*M* = 3.70, SD = 0.17) groups (*t* = 1.97, *p* = 0.067, 95% CI = –0.01 to –0.29) (Figure 1B). We observed that the variance was statistically different between groups for *OXTR* expression (Levine’s test for Equality of Variances: *F* = 7.87, *p* = 0.013). Therefore we conduced a two-sample *t*-test with equal variances not assumed and found that the difference between groups remained statistically significant for *OXTR* (*t* = 3.68, *p* = 0.004). The variance was not statistically difference between groups for *AVPR1A* expression (Levine’s test for Equality of Variances: *F* = 0.42, *p* = 0.53). Combined, these data suggest that abnormal function of OT system in WS, may in part be influenced by overexpression of *OXTR*. However, these data do not provide any information in terms of factors that may alter OT and AVP gene expression levels in WS.

## COMBINED MODEL: ALTERED DNA METHYLATION AND EXPRESSION OF OT AND AVP GENES IN WS

Gene expression is affected by many biochemical processes including transcription factors and DNA methylation. DNA methylation occurs when a methyl group forms a covalent attachment with the 5′ carbon of cytosine in the context of a cytosine phosphodiester guanine (CpG) dinucleotide, commonly called a CpG site. DNA methylation regulates gene expression by influencing the recruitment and binding of regulatory protein to DNA. Typically, an increase in DNA methylation at gene promoter regions is associated with a decrease in expression of that gene (Bonasio et al., 2010). Thought of in a different way; reduced DNA methylation is typically associated with an increase in gene expression.

As with the majority of biochemical processes, DNA methylation is regulated by a set of specific genes. For example, DNMT genes (e.g., *DMNT1*, *DMNT3a*, *DMNT3b*) code for the production of an enzyme called methyltransferase (Robertson et al., 1999). Methyltransferase functions to transfer a methyl group during DNA methylation. Disruption of *DMNT1* is associated with reduced levels of DNA methylation (Li et al., 1992) and an over expression of other genes (Rhee et al., 2002). Together, this leads to a novel question: are there any genes within the “WS classic deletion region” implicated to regulate DNA methylation? Identifying such genes may help to identify processes that impact gene expression within the OT and AVP systems in WS.

One gene commonly deleted in WS (*WBSCR22*) is involved in the production of methyltransferase (Doll and Grzeschik, 2001; Merla et al., 2002). Williams–Beuren syndrome chromosome region 22 (*WBSCR22*) is a gene deleted in WS that encodes a protein containing a nuclear localization signal and an *S*-adenosyl-L-methionine binding motif typical of methyltransferases. Together, the deletion *WBSCR22* in WS may affect DNA methylation and ultimately the expression of other genes.

**FIGURE 2:**
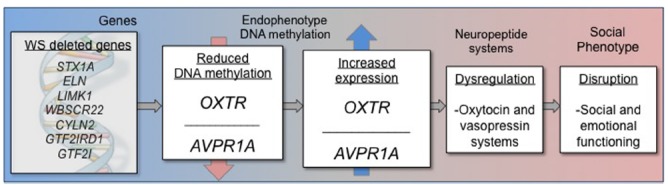
**Schematic representation of epigenetic model describing DNA methylation and gene expression impacting altered social behavior in Williams syndrome**.

The cause of disrupted OT and AVP functioning in many psychiatric conditions, including WS, is currently not known. In addition, it is not known if disrupted OT and AVP functioning is either a cause or an effect of altered social behavior and emotion processing in many conditions. Gene expression is regulated by a variety of biochemical processes, and it is reasonable to hypothesize that one of the genes in the WS deletion region regulates OT and AVP. Indeed the genes in the WS deletion region have widespread regulatory functions, and further characterization of the genes in this region will be vital for understanding how this deletion results in the cognitive and social characteristics typical in WS patients. The preliminary data presented here suggest that altered social behavior in WS may be influenced by overexpression of *OXTR* (Figure 2), which may, in turn, result in dysregulation of the OT systems.

## IMPACT AND FUTURE DIRECTIONS

A fundamental goal of behavioral genetics, neuroscience and psychology research is to understand how biological and mental processes are perturbed. One useful approach to elucidating genetic effects on abnormal functioning is to use “knock-out” animal models. Studying genetic effects on behavior in animals provides useful insight onto how specific genes impact other genetic, biochemical, or psychological functions in animals. However, this approach is extremely limited in terms of how findings from animals can be translated to the complex array of psychosocial processes that humans possess.

Empirical investigations of WS syndrome provide a unique opportunity to overcome this challenge. WS is caused by the deletion of a specify set of genes. Thus, studying WS holds the novel potential to associate the deletion of genes with downstream effects on genetic, biochemical and/or psychological systems in humans. Investigations of the genes of the WS deletion region or genes implicated in the social symptoms common in WS patients, including *OXTR* and *AVPR1A*, may only be observable in select tissues. Gene expression supports tissue-specific functions, and genes required for regulation of social function may only be characterized in brain regions that support social function or in relevant cell lines.

The pathophysiology underlying disrupted social-emotional processing in WS mirrors the pathophysiology often associated with other neurodevelopmental conditions such as autism (Tager-Flusberg et al., 2006). WS is characterized by an abnormally increased drive toward social interaction, while autism is characterized by a relative aversion toward social interaction. These opposing tendencies in social-emotional processing are also represented within the brain. For example, we have shown that WS is characterized by abnormally increased amygdala volume (Haas et al., 2014b), while autism is characterized by abnormally reduced amygdala volume (Nacewicz et al., 2006). Additionally (as directly related to our current hypotheses), WS is characterized by abnormally increased levels of OT (Dai et al., 2012), while autism is characterized by abnormally decreased levels of OT (Modahl et al., 1998). Lastly, there is compelling evidence of common genetic mechanisms influencing WS and autism. Specifically, while WS is caused by a deletion of genes at 7q11.23, the duplication of genes at 7q11.23 is associated with the presence of autistic symptoms (Sanders et al., 2011).

Thus, studying key systems influencing disrupted social behavior and emotional processing in WS has the important potential to improve our understanding of the pathophysiology of other neurodevelopmental conditions and in particular, autism. Interestingly, recent evidence indicates that autism is associated with abnormally increased DNA methylation of *OXTR* (Gregory et al., 2009). Based on the apparent “mirroring” of phenotypes between WS and autism, we predict to observe WS to be associated with abnormally *decreased* methylation of *OXTR*. Together, obtaining evidence that the deleted genes in WS influence the expression of OT genes will provide substantial insight onto genetic and biochemical factors that impact altered OT function in other neurodevelopmental conditions.

These preliminary findings have a clear and direct potential to improve treatment techniques for individuals with WS. For example, there is evidence that altering OT levels impacts sociability and communication in children and teens with autism. The results of these clinical trials, in combination with the potential results from this study, may contribute to the design of treatment approaches that target the OT or AVP systems in WS.

### Conflict of Interest Statement

The authors declare that the research was conducted in the absence of any commercial or financial relationships that could be construed as a potential conflict of interest.

## Footnotes

1 ncbi.nlm.nih.gov/geo/

2 ncbi.nlm.nih.gov/geo/info/geo2r.html
